# Is Recovery of Somatosensory Impairment Conditional for Upper-Limb Motor Recovery Early After Stroke?

**DOI:** 10.1177/1545968320907075

**Published:** 2020-05-11

**Authors:** Sarah B. Zandvliet, Gert Kwakkel, Rinske H. M. Nijland, Erwin E. H. van Wegen, Carel G. M. Meskers

**Affiliations:** 1 Department of Rehabilitation Medicine, Amsterdam Neuroscience and Amsterdam Movement Sciences, Amsterdam UMC, Vrije Universiteit Amsterdam, Amsterdam, Netherlands; 2 Department of Physical Therapy and Human Movement Sciences, Northwestern University, Chicago, IL, USA; 3 Department of Neurorehabilitation, Amsterdam Rehabilitation Research Centre, Reade, Amsterdam, Netherlands

**Keywords:** stroke, somatosensory disorders, motor activity, recovery of function, upper extremity

## Abstract

*Background*. Spontaneous recovery early after stroke is most evident during a time-sensitive window of heightened neuroplasticity, known as spontaneous neurobiological recovery. It is unknown whether poststroke upper-limb motor and somatosensory impairment both reflect spontaneous neurobiological recovery or if somatosensory impairment and/or recovery influences motor recovery. *Methods*. Motor (Fugl-Meyer upper-extremity [FM-UE]) and somatosensory impairments (Erasmus modification of the Nottingham Sensory Assessment [EmNSA-UE]) were measured in 215 patients within 3 weeks and at 5, 12, and 26 weeks after a first-ever ischemic stroke. The longitudinal association between FM-UE and EmNSA-UE was examined in patients with motor and somatosensory impairments (FM-UE ≤ 60 and EmNSA-UE ≤ 37) at baseline. *Results*. A total of 94 patients were included in the longitudinal analysis. EmNSA-UE increased significantly up to 12 weeks poststroke. The longitudinal association between motor and somatosensory impairment disappeared when correcting for progress of time and was not significantly different for patients with severe baseline somatosensory impairment. Patients with a FM-UE score ≥18 at 26 weeks (n = 55) showed a significant positive association between motor and somatosensory impairments, irrespective of progress of time. *Conclusions*. Progress of time, as a reflection of spontaneous neurobiological recovery, is an important factor that drives recovery of upper-limb motor as well as somatosensory impairments in the first 12 weeks poststroke. Severe somatosensory impairment at baseline does not directly compromise motor recovery. The study rather suggests that spontaneous recovery of somatosensory impairment is a prerequisite for full motor recovery of the upper paretic limb.

## Introduction

Somatosensory impairment is common in the acute phase after stroke, with prevalence rates between 34% and 84%,^1-4^ and is associated with reduced upper-limb motor function, activity, and participation poststroke; it is also related to increased hospital length of stay.^3-6^ The relation between motor and somatosensory impairments in the first 3 months poststroke may reflect parallel recovery in both modalities, driven by a common underlying neurobiological mechanism that occurs in a time-sensitive window of heightened neuroplasticity early after stroke,^7-10^ known as spontaneous neurobiological recovery.^8,9^ A number of clinical observational studies, however, suggest that severe somatosensory impairment may hamper motor recovery poststroke.^11,12^ This relationship may specifically be explained by the importance of somatosensory input for fine motor skills of the upper limb.^12^

Previous prospective studies found that spontaneous neurobiological recovery, as reflected by progress of time alone, is the most significant covariate for explaining the recovery pattern of neurological impairments in the first 8 to 10 weeks poststroke.^7^ In addition, a number of observational studies indicated that spontaneous neurobiological recovery is proportional to initial upper-limb,^13-15^ lower-limb,^16,17^ and somatosensory impairments^18,19^; aphasia^20,21^; and visual spatial neglect (VSN)^21,22^, with a recovery range between 64%^16,19^ and 97%.^22^ Patients who failed to show spontaneous recovery of VSN after a first-ever ischemic right hemispheric stroke also have a high probability to fail recovery on other affected modalities such as motor impairment of the upper paretic limb (ie, so-called non-recoverers of spontaneous neurobiological recovery).^22^ Nijboer et al^23^ showed that less improvement on the Fugl-Meyer motor assessment of the Upper Extremity (FM-UE) was independently associated with more severe VSN in the first 10 weeks poststroke, suggesting a suppressive effect of neglect on upper-limb motor recovery within the time window of spontaneous neurobiological recovery.^23^ Finally, evidence was found that patients who did not show a pattern of spontaneous neurobiological recovery in their lower limb are also not likely to show upper-limb recovery within the first 6 months poststroke.^16^ These results suggest that poststroke recovery is driven by common underlying processes reflected by spontaneous neurobiological recovery, spanning multiple modalities.^9,13-16,18-22^ Multiple processes such as salvation of penumbral tissue,^24^ upregulation of growth promoting factors, gene-dependent enhancement of angiogenesis,^25^ and alleviation of diaschisis^24^ are mentioned as factors that may drive spontaneous neurobiological recovery. Unfortunately, the above-mentioned mechanisms are still poorly understood, and no causal marker has yet been identified that can accurately predict who will or will not show spontaneous neurobiological recovery early after stroke.^8,9,26,27^

Meyer et al^4^ previously showed in a cross-sectional study in 122 patients within the first 6 months poststroke that motor and somatosensory impairments are low to moderately correlated (*r* = 0.22-0.61). To further disentangle the relationship between motor and somatosensory recovery, i.e., whether both can be explained from general mechanisms of spontaneous neurobiological recovery or if somatosensory impairment and/or recovery influences motor recovery, a longitudinal study is required. In addition, the absence of somatosensory input could compromise experience-dependent plasticity, which underlies the remodeling of neural circuits and could, therefore, impair the development of new motor programs after stroke.^28-31^ In this latter situation, one expects a failure in recovery of somatosensory impairment to be significantly associated with less motor recovery of the upper paretic limb.

In the present study, we aimed to describe the time course of somatosensory recovery and to analyze the longitudinal association between motor and somatosensory impairments in the first 6 months poststroke. We examined if the association between motor and somatosensory impairments remained after adjusting for progress of time, as a reflection of spontaneous neurobiological recovery,^7^ and whether this longitudinal association was different in patients with an initially severe baseline level of somatosensory impairment when compared with those with a mild to moderate sensory impairment in the first week poststroke. Finally, we aimed to investigate whether the association between motor and somatosensory impairments depend on the presence of motor recovery of the upper paretic limb. For this latter aim, we investigated the difference between patients who showed motor recovery (ie, recoverers) compared with those who did not show spontaneous motor recovery of the upper limb (ie, non-recoverers) in the first 6 months poststroke.

## Materials and Methods

Data were derived from 3 longitudinal studies: the EXPLICIT,^32^ EXPLORE-stroke, and 4D-EEG cohorts, with a total of 215 patients. The EXPLICIT randomized controlled trial investigated the effects of a modified constraint-induced movement therapy (mCIMT) and EMG-triggered neuromuscular stimulation (EMG-NMS) on stroke recovery mechanisms compared with usual care (Trial NL1366, NTR1424). Patients were included within 3 weeks poststroke and assessed weekly during the first 5 weeks and then at 8, 12, and 26 weeks poststroke.^33^ Voluntary finger extension was used to stratify patients into a group with a favorable prognosis for upper-limb motor recovery, who received mCIMT or usual care, and a group with an unfavorable prognosis, who received EMG-NMS or usual care. Neither mCIMT nor EMG-NMS significantly influenced upper-limb motor recovery in terms of FM-UE at any time point in the first 6 months poststroke.^32^ Hence, the present study used data of the total sample. Patients enrolled in the EXPLORE-stroke or 4D-EEG cohort studies all received usual care following the current Dutch Guidelines of Physiotherapy.^34^

EXPLORE-stroke and 4D-EEG (Trial NL4084, NTR4221) were longitudinal observational cohort studies that both assessed clinical scales as well as neurophysiological parameters in a repetitive manner to improve prediction models and enhance understanding of functional recovery after stroke. In line with recent recommendations,^8^ clinical assessments in the EXPLORE-stroke and 4D-EEG studies were made at fixed times poststroke—that is, within 3 weeks and at 5, 12, and 26 weeks poststroke. Patients in the 4D-EEG study were additionally assessed at 8 weeks poststroke.

Within the aforementioned cohorts, the following inclusion criteria were used: (1) having experienced a first-ever, ischemic hemispheric stroke, verified by CT and/or MRI scan less than 3 weeks before inclusion; (2) having an upper-limb paresis as defined by a National Institute of Health Stroke Scale (NIHSS) score of 1 or more; (3) being aged between 18 and 80 years; (4) having no severe cognitive deficits (Mini Mental State Examination of at least 19 points)^35,36^; (5) being able to sit for 30 s without support; (6) having no orthopedic limitations of the upper limb; and (7) having no preexisting neurological condition. All procedures were in accordance with the declaration of Helsinki and were approved by the Medical ethics committees of Leiden University Medical Center (EXPLICIT: NL21396.058.08; EXPLORE-stroke: NL39323.058.12) or VU University Medical Center (4D-EEG: NL47079.029.14). All participants gave their written informed consent.

### Measuring Somatosensory Impairment and Determining Baseline Level of Impairment

Somatosensory impairment of the upper extremity was assessed using the Erasmus MC modification of the (revised) Nottingham Sensory Assessment (EmNSA).^37^ The intrarater and interrater reliability of the EmNSA for the upper limb are predominantly good to excellent (κ = 0.62-1.00 intrarater and κ = 0.48-1.00 interrater reliability) for patients with intracranial disorders.^37^ The EmNSA uses a 3-point ordinal scale and offers a reliable somatosensory assessment of the upper and lower limbs for patients with intracranial disorders. The testing procedure includes a pinprick test to assess tactile sensation, sharp-blunt discrimination to assess pain sensation, and measuring proprioception to assess gnostic sensibility. The maximum score of the EmNSA for the upper extremity (EmNSA-UE) is 40 points. A score of 39 points or lower has been described as a somatosensory impairment^37^; because the measurement error of the EmNSA-UE has not been established, we considered a baseline score <38 points as indicating somatosensory impairment, accounting for a measurement error of 5%.

### Defining Baseline Level of Impairment Following EmNSA-UE

Patients were categorized as having high and low baseline scores on the EmNSA-UE to differentiate between severe and moderate somatosensory impairments (baseline EmNSA-UE level). To distinguish between these groups, a dichotomous variable was constructed based on the NIHSS item score of somatosensory impairment at 26 weeks poststroke, distinguishing between having no somatosensory impairment (0 points) and having a somatosensory impairment (1 or 2 points) as the state variable in the receiver operating characteristic (ROC) curve.^38^ The cutoff for low and high baseline scores on the EmNSA-UE within 3 weeks poststroke was determined by inspecting the ROC curve, in which an optimum between sensitivity and specificity was sought, prioritizing sensitivity.

### Measuring Motor Impairment, Determining Grouping Variables: Baseline Level of Impairment and Recovery Patterns

Motor impairment of the upper limb was measured with the FM-UE.^39,40^ To account for a 6 point measurement error, patients were considered to have a motor impairment when the baseline score was 60 (out of the 66) points or less.^41^

### Defining Baseline Level of Impairment Following FM-UE

The baseline level for severe motor impairment was set at a cut-off of 18 points on the FM-UE.^9,13,14^ The 18-point FM-UE cutoff derived from Winters et al^14^ was checked for suitability for the current study by constructing a ROC curve. The NIHSS item on motor impairment of the affected upper limb was used to construct the state variable, after which the same steps were applied as those described for somatosensory impairment level.

### Defining the Recovery Pattern Subgroups: Recoverers and Non-recoverers Following FM-UE

In the case of a severe baseline level of motor impairment, a further distinction in motor recovery pattern subgroups was made. This distinction between ‘recoverers’ and ‘non-recoverers’ was made based on whether or not a patient showed clinically relevant improvement^41^ on the FM-UE over time as an indication of spontaneous neurobiological recovery, and was defined as:

- Non-recoverers: FM-UE score <18 at baseline, <6 points improvement or FM-UE score <18 points at 26 weeks poststroke
- Recoverers: FM-UE score <18 points at baseline with ≥6 points improvement resulting in ≥18 points at 26 weeks poststroke

### Measuring Covariates

Covariates that are assumed to affect or are associated with sensorimotor recovery of the upper limb,^14,23,42,43^ were considered as possible confounders in the longitudinal association between motor and somatosensory recovery. These were as follows: (1) age; (2) affected hemisphere; (3) comorbidities, measured with the Cumulative Illness Rating Score (CIRS)^44^; (4) visuospatial neglect, assessed with a single-target Letter Cancellation Test (LCT^45^; Patients were instructed to mark all O’s on an A4 sheet, which was aligned to the patient’s sagittal midline. The sheet showed 20 O’s on both sides of the midline, mixed with random letters. The marked O’s in the contralesional visual field were counted); (5) Motor impairment of the lower limb, measured with the Motricity Index of the lower extremity (MI-LE)^46^; and (6) stroke severity, longitudinally measured with the National Institutes of Health Stroke Scale (NIHSS).^47^ This scale evaluates the severity of possibly affected modalities after stroke. To account for overlaps with FM-UE, EmNSA-UE, LCT, and MI-LE, a NIHSS-adapted variable was constructed by leaving out items that measure upper- and lower-limb motor impairment, limb ataxia, somatosensory impairment, extinction, and inattention (items 5a, 5b, 6a, 6b, 7, 8, and 11).

### Statistical Analysis

The time course of somatosensory recovery was described using a mixed-model analysis, with the EmNSA-UE scores from baseline until 26 weeks poststroke for patients with both a motor and sensory impairment, based on the abovementioned cutoff values. The longitudinal association between motor (FM-UE) and somatosensory impairments (EmNSA-UE) was analyzed using a second association model.

To evaluate if this association was robust for confounders, we examined the influence of covariates: age, lesion side, CIRS, NIHSS-adapt, LCT, and MI-LE. An adjustment for time was subsequently made to determine if the association was partly independent of progress of time and, therefore, of spontaneous neurobiological recovery. We further evaluated the interaction effect of severity of somatosensory impairment at baseline on the longitudinal association of recovery between motor and somatosensory impairments.

We investigated whether the association between motor and somatosensory recovery differed for patients with a low versus a high baseline score on the FM-UE and for recoverers versus non-recoverers. We investigated this using 2 different methods. For the first method, we constructed dichotomous grouping variables based on the baseline FM-UE level and the FM-UE recovery pattern subgroups, as described above—that is, recoverers and non-recoverers. Interaction terms between the baseline FM-UE level and EmNSA-UE and between FM-UE recovery patterns and EmNSA-UE were added and evaluated for statistical significance. Second, as an alternative for defining grouping variables, an alternative model was used in which within- and between-subject effects are separated.^48^ This, so-called hybrid model enables a direct distinction between factors relating to differences in recovery within a patient over time and factors relating to differences in recovery between patients.^48^ For this purpose, we calculated the mean EmNSA-UE score for each individual patient, representing the between-subject part of the association, and the EmNSA-UE scores per measurement moment minus the patient’s mean score, representing the within-subject part. The association between FM-UE and EmNSA-UE was reanalyzed, estimating 2 separate β-coefficients to represent the within- and between-subject parts of the association.

To correct for dependence between measurements, a random intercept per patient was used in the models. Residuals were checked for normality by inspection of the probability distributions (q-q plots) and histograms. Significance level was set at a 2-tailed α of .05 for all analyses. All analyses were performed using IBM SPSS statistics version 22 (IBM corporation, Armonk, NY).

## Results

**Figure 1.**
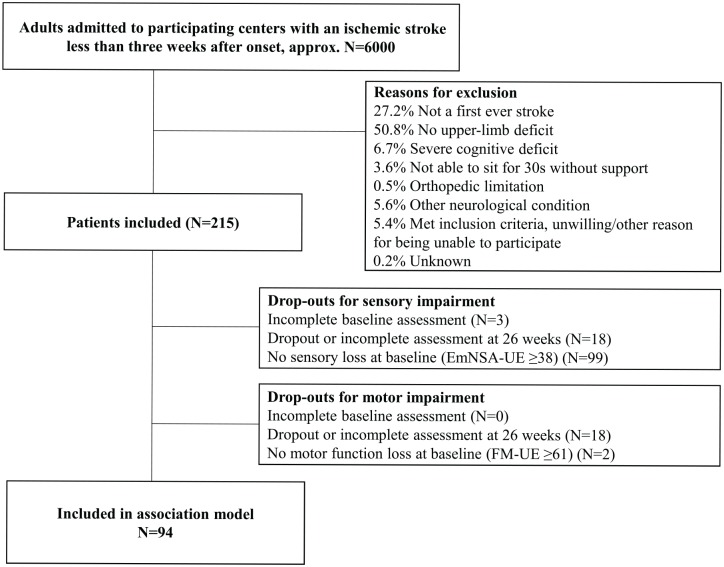
Flowchart of the included patients with a stroke. Abbreviations: EmNSA-UE, Erasmus modification of the Nottingham Sensory Assessment of the Upper Extremity; FM-UE, Fugl-Meyer Motor Assessment of the Upper Extremity.

The flowchart of patient inclusion is shown in Figure 1. Participant characteristics are displayed in Table 1. Data of 215 patients were collected within the 3 abovementioned studies between October 2008 and May 2017. Of the 197 patients from whom sufficient data were collected, 195 patients had a motor impairment—that is, an FM-UE score of 60 points or less at baseline. A total of 95 patients (49.0% of the patients with a complete data set) had an initial somatosensory impairment following the EmNSA-UE score of 37 points or less at baseline. Combining both modalities, data of 94 patients were available to determine the longitudinal association. All residuals of the mixed model analysis were normally distributed.

**Table 1.** Participant Characteristics.^a^

Characteristic	All Participants	High Baseline Motor Score	Low Baseline Motor Score, Recoverers	Low Baseline Motor Score, Non-recoverers
	n = 94	n = 34	n = 21	n = 39
Time between stroke and baseline measurements (days)^b^	9.6 (4.7)	10.7 (5.0)	10.0 (4.8)	8.3 (4.1)
Age (year)^b^	60.3 (12.5)	60.6 (14.3)	62.1 (10.9)	59.1 (11.8)
Gender, male/female (n)^c^	58/34	21/13	12/9	25/14
Affected hemisphere, left/right/(n)^c^	27/67	10/24	9/12	8/31
Bamford classification, LACI, PACI, or TACI (n)^c^	32/53/9	14/18/2	8/11/2	10/24/5
CIRS	2 (2-4)	3.5 (2-5.25)	2 (2-4)	2 (1-4)
NIHSS	9 (5-12)	5 (3.75-7)	8.5 (8-10)	12 (10-13)
LCT at baseline	14 (3-19)	19 (14-20)	17.5 (13.25-20)	4 (0-14)
LCT at 6 months PS	19.5 (17-20)	20 (19-20)	20 (18.25-20)	19 (15-20)
FM-UE at baseline	7 (4-30)	35 (26.25-47.5)	7 (5.5-8.5)	4 (2-5)
FM-UE at 6 months PS	24 (7.75-57)	58.5 (49-62.25)	33 (22-52.5)	7 (5-9)
ARAT at baseline	0 (0-6.5)	16 (6-27.5)	0 (0-0)	0 (0-0)
ARAT at 6 months PS	10.5 (0-43.25)	49.5 (37.75-55)	22 (7-39.5)	0 (0-0)
EmNSA-UE at baseline	24 (2-34)	32 (10.75-36)	32 (4.5-35.5)	6 (0-25)
EmNSA-UE at 6 months PS	39.5 (24.25-40)	40 (36.75-40)	40 (36.5-40)	35 (14-40)
MI-UE at baseline	11 (0-49.5)	58 (47-65)	12.5 (0-28.75)	0 (0-0)
MI-UE at 6 months PS	47 (18-76)	84 (76-92)	65 (47-76)	14 (0-28)
MI-LE at baseline	42 (9-64)	75 (53-100)	42 (28-56.75)	9 (0-23)
MI-LE at 6 months PS	69 (47-89)	100 (77.5-100)	72 (64-75)	43 (37-64)

Abbreviations: ARAT, Action Research Arm Test; CIRS, Cumulative Illness Rating Scale; EmNSA-UE, Erasmus modification of the Nottingham Sensory Assessment of the Upper Extremity; FM-UE, Fugl-Meyer Motor Assessment of the Upper Extremity; LACI, lacunar anterior circulation infarct; LCT, Letter Cancellation Test; LE, lower extremity; MI, Motricity Index; NIHSS, National Institute of Health Stroke Scale; PACI, partial anterior circulation infarct; PS, poststroke; TACI, total anterior circulation infarct; UE, upper extremity.

a FM-UE score of 18 points or higher is considered a high baseline score for motor impairment, a FM-UE score lower than 18 points is considered a low baseline score. Patients with an FM-UE score <18 points were divided into recoverers (FM-UE ≥18 points at 26 weeks and at least a 6-point improvement between baseline and 26 weeks PS) and non-recoverers (FM-UE <18 points at 26 weeks PS or failing to show a 6-point improvement between baseline and 26 weeks PS). Unless indicated otherwise, the provided scale is ordinal, and median and interquartile ranges are displayed. Baseline value is the first measurement of each subject within 3 weeks PS.

b Continuous variable; means and standard deviations are displayed.

c Categorical/nominal variable; number of patients is displayed.

### Time Course of Somatosensory Recovery

**Figure 2.**
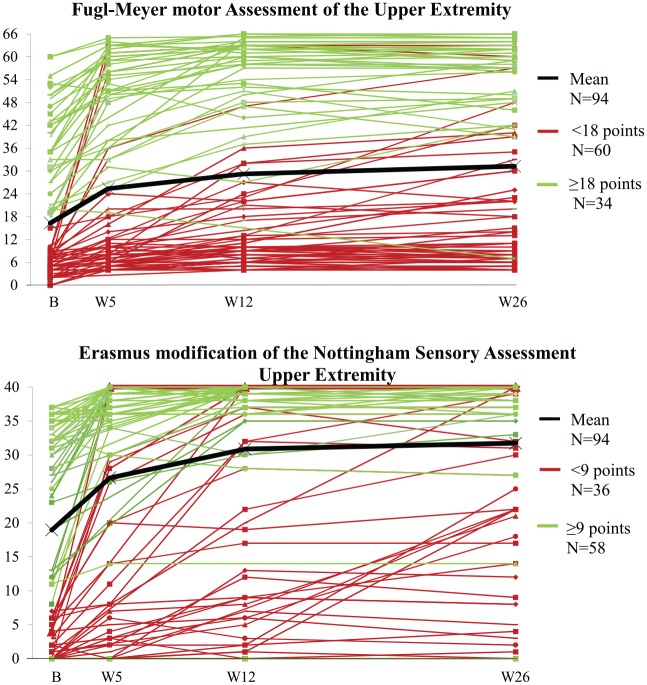
Time course of FM-UE and EmNSA-UE over the first 26 weeks poststroke.^a^ Abbreviations: B, baseline assessment within 3 weeks post-stroke; EmNSA-UE, Erasmus modification of the Nottingham Sensory Assessment of the Upper Extremity; FM-UE, Fugl-Meyer Motor Assessment of the Upper Extremity; W, week of measurement. ^a^A FM-UE score <18 points was considered a low baseline score and >18 points, a high baseline score. An EmNSA-UE score <9 points was considered a low baseline score and >9 points, a high baseline score.

Figure 2 shows the individual time courses of motor and somatosensory recovery for the 94 patients included in the association model up to 26 weeks poststroke. Mean EmNSA-UE increased significantly up to week 12 poststroke. Although no further significant increase was found between 12 and 26 weeks at a group level, there is evidence of change at an individual level, as can be seen in Figure 2. Table 2 displays the corresponding descriptives and the β estimates with 95% CIs and probability values of the association model describing the time course of somatosensory recovery.

**Table 2.** Measurement Descriptives and Model Outcomes: Association Model of EmNSA-UE and Time.^a^

		Descriptives Per Measurement					
		Baseline	W5	W12	W26		
EmNSA-UE							
Median		24	36	39	39.5		
IQR		[2-34]	[8-39]	[23.5-40]	[24.5-40]		
FM-UE							
Median		7	12	20	24		
IQR		[4-30]	[5-51]	[8-58]	[7.75-57]		
	Association Model of EmNSA-UE and Time						
EmNSA-UE	Baseline	Base to W5	Base to W12	Base to W26	W5 to W12	W5 to W26	W12 to W26
β	19.0	7.8	11.2	12.5	3.5	4.8	1.3
CI	[16.1-21.9]	[6.0-9.6]	[9.5-13.1]	[10.8-14.3]	[1.6-5.3]	[2.9-6.6]	[−0.5-3.1]
*P*	—	<.01	<.01	<.01	<.01	<.01	.15

a Abbreviations: EmNSA-UE, Erasmus modification of the Nottingham Sensory Assessment of the Upper Extremity; FM-UE, Fugl-Meyer motor assessment of the Upper Extremity; IQR, interquartile range; W, week of measurement; β, Beta estimate; CI, confidence interval; *P* value, probability value for the tested model.

Base: baseline, that is, the first measurement of each patient within 3 weeks poststroke; low base: baseline FM-UE score below 18; high base: baseline FM-UE score 18 or higher; recoverers: FM-UE 18 or higher at 26 weeks poststroke and at least a 6-point improvement between baseline and 26 weeks poststroke; non-recoverers: that is, FM-UE below 18 at 26 weeks poststroke or failed to show 6-point improvement between baseline and 26 weeks poststroke.

### Longitudinal Association of Motor and Somatosensory Impairments and Impact of Progress of Time

**Figure 3.**
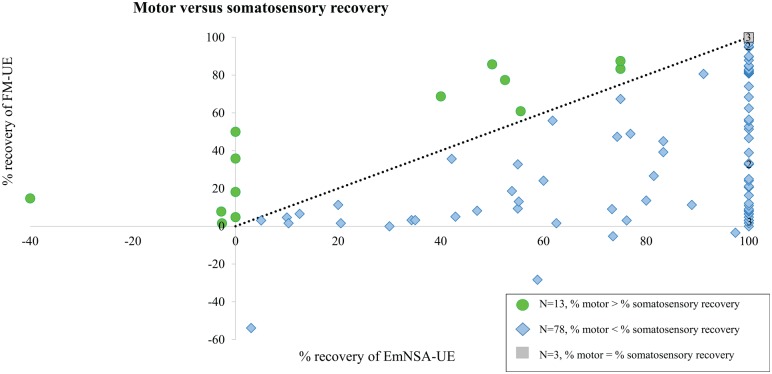
Percentage of motor and somatosensory recovery between baseline (within 3 weeks poststroke) and 26 weeks poststroke.^a^ ^a^Abbreviations: EmNSA-UE, Erasmus modification of the Nottingham Sensory Assessment of the Upper Extremity; FM-UE, Fugl-Meyer Motor Assessment of the Upper Extremity. Motor and somatosensory recovery of 94 patients expressed as a percentage of the maximum possible improvement: EmNSA-UE recovery = (EmNSA-UE 26 weeks/[40 − EmNSA-UE baseline]) × 100%; FM-UE recovery = (FM-UE 26 weeks/[66 − FM-UE baseline]) × 100%. The black dashed line represents the same percentage recovery of both modalities. When patients show relatively more somatosensory than motor recovery, their value (blue diamond) is below the dashed line; n = 78. When patients show relatively more motor than somatosensory recovery, their value (green dot) is above the dashed line; n = 13. Three patients showed 100% recovery of both modalities (gray square at the top corner).

Figure 3 shows the recovery of motor and somatosensory impairments expressed as a percentage of the maximum possible recovery on the FM-UE and EmNSA-UE, as a visual illustration of their relationship. Most patients (n = 78) showed relatively more somatosensory than motor recovery; but 13 patients (14%) showed more motor than somatosensory recovery. Table 3 displays the outcome of the association model between motor and somatosensory impairments. FM-UE and EmNSA-UE showed a significant longitudinal association (β intercept = 10.91; β EmNSA-UE = 0.55; *P* < .01). The longitudinal association between FM-UE and EmNSA-UE changed, yet remained significant when adjusting for age, lesion side, CIRS, NIHSS-Adapt, LCT, and MI-LE, with β intercept =3.42 and β EmNSA-UE =0.21; *P* < .01. The corresponding CIs are listed in Table 3. The longitudinal association between FM-UE and EmNSA-UE was no longer significant after adjusting for progress of time.

**Table 3.** Measurement Descriptives and Model Outcomes: Association Models of FM-UE and EmNSA-UE.^a^

Association Model of FM-UE and EmNSA-UE											
FM-UE	Constant	EmNSA									
β	10.91	0.55									
CI	[5.78-16.04]	[0.43-0.67]									
*P*	—	<.01									
Association Model of FM-UE and EmNSA-UE Adjusted for Covariates											
FM-UE	Constant	EmNSA	Age	Lesion Side Right	CIRS	NIHSS-Adapt	LCT	MI-LE			
β	3.42	0.21	0.02	−3.44	1.90	−2.20	−0.31	0.34			
CI	[−13.38-20.21]	[0.06-0.35]	[−0.22-0.26]	[−10.49-3.61]	[0.51-3.29]	[−3.81-−0.60]	[−0.62-0.00]	[0.26-0.42]			
*P*	—	<.01	.86	.33	<.01	<.01	.05	<.01			
Association Model of FM-UE and EmNSA-UE Adjusted for Covariates and Time											
FM-UE	Constant	EmNSA	Age	Lesion Side Right	CIRS	NIHSS-Adapt	LCT	MI-LE	Base to W5	Base to W12	Base to W26
β	9.81	0.13	−0.02	−4.11	2.15	−1.02	−0.45	0.21	5.60	7.83	9.06
CI	[−10.41-30.03]	[−0.03-0.28]	[−0.31-0.28]	[−12.81-4.60]	[0.40-3.90]	[−2.71-0.68]	[−0.76-−0.15]	[0.12-0.30]	[2.01-9.19]	[3.72-11.95]	[4.83-13.29]
*P*	—	.10	.91	.35	.02	.24	<.01	<.01	<.01	<.01	<.01

^a^Abbreviations: CIRS, Cumulative Illness Rating Score; EmNSA-UE, Erasmus modification of the Nottingham Sensory Assessment of the Upper Extremity; FM-UE, Fugl-Meyer motor assessment of the Upper Extremity; LCT, Letter Cancellation Task; MI-LE, Motricity Index for Lower Extremity; NIHSS-Adapt, National Institute of Stroke Scale without items 5a, 5b, 6a, 6b, 7, 8, and 11; W, week of measurement; β, Beta estimate; CI, confidence interval; P value, probability value for the tested model.

Base: baseline, that is, the first measurement of each patient within 3 weeks poststroke; low base: baseline FM-UE score lower than 18; high base: baseline FM-UE score 18 or higher; recoverers: FM-UE 18 or higher at 26 weeks poststroke and at least a 6-point improvement between baseline and 26 weeks poststroke; non-recoverers: that is, FM-UE lower than 18 or lower at 26 weeks poststroke or failed to show 6-point improvement between baseline and 26 weeks poststroke.

### Categorized Baseline Level of Somatosensory Impairment

The ROC curve for somatosensory impairment showed an optimal cutoff at 9 points on the EmNSA-UE. Of the 95 patients with a somatosensory impairment at baseline, 37 (39.0%) had an EmNSA-UE score lower than 9 points and were categorized as having a severe somatosensory impairment. Eight patients had a low baseline score on the EmNSA-UE while having a high baseline score on the FM-UE. In all, 32 patients had a high baseline score on the EmNSA-UE while having a low baseline score on the FM-UE. The remaining 54 patients showed either high (26 patients) or low (28 patients) baseline scores for both modalities.

### Influence of Severe Baseline Somatosensory Impairment on Motor Recovery

The longitudinal association between motor and somatosensory impairments did not differ significantly between patients with a high or low level of somatosensory impairment at baseline since no significant interaction effect was found between longitudinal EmNSA-UE score and baseline EmNSA-UE level; *P* = .09.

### Categorized Baseline Level of Motor Impairment: Recoverers and Non-recoverers

The ROC curve for motor impairment confirmed the optimal cutoff point of 18 points on the FM-UE. Of the 195 patients with a motor impairment at baseline, 109 (55.9%) had a FM-UE score lower than 18 points and were categorized as having a severe motor impairment, of whom 60 were included in the analyses because of a somatosensory impairment. Of these 60 patients with a low baseline score, 21 were categorized as recoverers and 39 as non-recoverers based on their FM-UE score at 26 weeks poststroke.

### Influence of Somatosensory Recovery on Motor Recovery, High Versus Low Baseline and Recoverers Versus Non-recoverers

A significant interaction effect was found between longitudinal EmNSA-UE and baseline FM-UE levels. For the group with a high baseline score (FM-UE ≥ 18), this resulted in a significant longitudinal association between FM-UE and EmNSA-UE: β intercept = 2.75; β EmNSA-UE = 0.44; *P* < .01. Among the group with a low FM-UE baseline score, no significant association was found between longitudinal FM-UE and EmNSA-UE scores.

A significant interaction effect was also found between longitudinal EmNSA-UE and motor recovery pattern, with a significant positive association for the recoverers—β intercept = 3.97; β EmNSA-UE = 0.69; *P* < .01—whereas the non-recoverers showed a negative association: β intercept = 11.29; β EmNSA-UE = −0.16; *P* = .02. Table 3 gives all corresponding values of the association model between motor and somatosensory impairments for the groups based on baseline level of motor impairment and motor recovery pattern.

**Table:** Measurement Descriptives and Model Outcomes: Association Models of FM-UE and EmNSA-UE, With Interaction Effects of Baseline FM-UE Level and Recovery Pattern Groups.^a^

Association Model of FM-UE and EmNSA-UE, for Low/High FM-UE Score at Baseline, Adjusted for Covariates and Time													
FM-UE	Constant Low FM-UE Base	EmNSA Low FM-UE Base	Constant High FM-UE Base	EmNSA High FM-UE Base	Age	Lesion Side Right	CIRS	NIHSS-Adapt	LCT	MI-LE	Base to W5	Base to W12	Base to W26
β	2.75	0.05	22.99	0.44	0.08	−5.05	0.08	−0.91	−0.35	0.15	5.9	8.45	9.7
CI	[−10.11-15.61]	[−0.09-0.19]	[8.03-37.96]	[0.22-0.65]	[−0.10-0.26]	[−10.34-0.25]	[−1.00-1.16]	[−2.35-0.52]	[−0.61-−0.08]	[0.07-0.23]	[2.57-9.23]	[4.68-12.22]	[5.85-13.55]
*P*	—	.46	—	<.01	.36	.06	.89	.21	.01	<.01	<.01	<.01	<.01
Association Model of FM-UE and EmNSA-UE in n = 60 With a Low Baseline FM-UE Score, for Recoverers/Non-recoverers, Adjusted for Covariates and Time													
FM-UE	Constant Recoverers	EmNSA Recoverers	Constant Non-recoverers	EmNSA Non-recoverers	Age	Lesion Side Right	CIRS	NIHSS-Adapt	LCT	MI-LE	Base to W5	Base to W12	Base to W26
β	3.97	0.69	11.29	−0.16	−0.03	−3.62	0.18	−1.51	−0.26	0.07	4.85	7.71	9.25
CI	[−12.33-20.28]	[0.4-0.90]	[−2.74-25.31]	[−0.30-−0.03]	[−0.22-0.16]	[−9.02-1.78]	[−1.03-1.38]	[−2.96-−0.06]	[−0.51-−0.00]	[−0.03-0.16]	[1.07-8.63]	[3.07-12.36]	[4.46-14.04]
*P*	—	<.01	—	.02	.76	.19	.77	.04	.05	.15	.01	<.01	<.01

^a^Abbreviations: CIRS, Cumulative Illness Rating Score; EmNSA-UE, Erasmus modification of the Nottingham Sensory Assessment of the Upper Extremity; FM-UE, Fugl-Meyer motor assessment of the Upper Extremity; LCT, Letter Cancellation Task; MI-LE, Motricity Index for Lower Extremity; NIHSS-Adapt, National Institute of Stroke Scale without items 5a, 5b, 6a, 6b, 7, 8, and 11; W, week of measurement; β, Beta estimate; CI, confidence interval; *P* value, probability value for the tested model.

Base: baseline, that is, the first measurement of each patient within 3 weeks poststroke; low base: baseline FM-UE score lower than 18; high base: baseline FM-UE score 18 or higher; recoverers: FM-UE 18 or higher at 26 weeks poststroke and at least a 6-point improvement between baseline and 26 weeks poststroke; non-recoverers: that is, FM-UE lower than 18 at 26 weeks poststroke or failed to show 6-point improvement between baseline and 26 weeks poststroke.

### Influence of Somatosensory Recovery on Motor Recovery, Based on Between- and Within-Subject Effects

Table 4 shows the individual between- and within-subject effects of the association between motor and somatosensory impairments. Both the between- and within-subject parts showed a significant longitudinal association between FM-UE and EmNSA-UE: β intercept = 10.22; β EmNSA-UE/between = 0.57, *P* < .01, and β EmNSA-UE/within = 0.54, *P* < .01. When correcting for age, lesion side, LCT, CIRS, NIHSS-Adapt, and MI-LE, only the between-subject association between FM-UE and EmNSA-UE remained significant: β intercept = −1.15; β EmNSA-UE/between = 0.35, *P* < .01, and β EmNSA-UE/within = 0.12, *P* = .16. The between association increased after adjustment for progress of time: β intercept = −0.21; β EmNSA-UE/between = 0.49, *P* < .01, and β EmNSA-UE/within = 0.00, *P* = .98. No significant interaction effects were found between the baseline EmNSA-UE and FM-UE levels or the baseline EmNSA-UE level and the pattern of spontaneous motor recovery.

**Table 4.** Measurement Descriptives and Model Outcomes: Hybrid Association Models of FM-UE and EmNSA-UE.^a^

Hybrid Association Model of FM-UE and EmNSA-UE												
FM-UE	Constant	EmNSA/Between	EmNSA/Within									
β	10.22	0.57	0.54									
CI	[1.18-19.26]	[0.27-0.88]	[0.41-0.68]									
*P*	—	<.01	<.01									
Hybrid Association Model of FM-UE and EmNSA-UE Adjusted for Covariates												
FM-UE	Constant	EmNSA /Between	EmNSA /Within	Age	Lesion Side Right	CIRS	NIHSS-Adapt	LCT	MI-LE			
β	−1.15	0.35	0.12	0.02	−2.62	1.73	−2.34	−0.28	0.35			
CI	[−18.32-16.01]	[0.12-0.58]	[−0.05-0.30]	[−0.21-0.25]	[−9.57-4.33]	[0.36-3.10]	[−3.95-−0.74]	[−0.59-0.03]	[0.27-0.43]			
*P*	—	<.01	.16	.87	.45	.01	<.01	.08	<.01			
Hybrid Association Model of FM-UE and EmNSA-UE Adjusted for Covariates and Time												
FM-UE	Constant	EmNSA/Between	EmNSA/Within	Age	Lesion Side Right	CIRS	NIHSS-Adapt	LCT	MI-LE	Base to W5	Base to W12	Base to W26
β	−0.21	0.49	−0.00	−0.03	−2.11	1.78	−0.88	−0.45	0.20	7.03	9.87	11.29
CI	[−21.05-20.64]	[0.20-0.77]	[−0.18-0.18]	[−0.32-0.26]	[−10.71-6.48]	[0.06-3.51]	[−2.56-0.81]	[−0.75-−0.15]	[0.11-0.29]	[3.34-10.73]	[5.56-14.19]	[6.83-15.76]
*P*	—	<.01	.98	.83	.62	.04	.31	<.01	<.01	<.01	<.01	<.01

^a^Abbreviations: CIRS, Cumulative Illness Rating Score; EmNSA-UE, Erasmus modification of the Nottingham Sensory Assessment of the Upper Extremity; FM-UE, Fugl-Meyer motor assessment of the Upper Extremity; LCT, Letter Cancellation Task; MI-LE, Motricity Index for Lower Extremity; NIHSS-Adapt, National Institute of Stroke Scale without items 5a, 5b, 6a, 6b, 7, 8, and 11; W, week of measurement; β, Beta estimate; CI, confidence interval; *P* value, probability value for the tested model.

Base: baseline, that is, the first measurement of each patient within 3 weeks poststroke; low base: baseline FM-UE score lower than 18; high base: baseline FM-UE score 18 or higher; recoverers: FM-UE 18 or higher at 26 weeks poststroke and at least a 6-point improvement between baseline and 26 weeks poststroke; non-recoverers: that is, FM-UE lower than 18 at 26 weeks poststroke or failed to show 6-point improvement between baseline and 26 weeks poststroke.

## Discussion

In the present study, we investigated the longitudinal association between motor and somatosensory impairments in a cohort of 94 patients with a first-ever ischemic stroke, measured at 4 fixed time points during the first 6 months poststroke.

We show that motor recovery was significantly longitudinally associated with somatosensory recovery. Both modalities recover within the same time window of spontaneous neurobiological recovery in the first 3 months poststroke. After adjusting for possible confounders such as age and comorbidities, the association between motor and somatosensory impairment remained significant, underpinning its robustness. However, the association disappeared when correcting for progress of time, suggesting that time-dependent change resulting from spontaneous neurobiological recovery is the main factor that drives improvement of both modalities in the same time window.^7,49^ In the longitudinal association model between FM-UE and EmNSA-UE, we found no significant influence of baseline level of somatosensory impairment. The current findings, therefore, suggest that severe somatosensory impairment in the first weeks poststroke does not necessarily obstruct motor recovery.

However, we did find evidence that somatosensory function is an important factor to achieve full recovery of motor impairment. None of the patients with full motor recovery showed impaired somatosensory recovery. Our results suggest that different mechanisms are relevant in subgroups of patients who show or do not show spontaneous neurobiological motor recovery of the upper paretic limb. In patients with a low level of motor impairment at baseline (FM-UE > 18) and/or significant spontaneous improvement of motor impairment over time (ie, recoverers), an association between motor and somatosensory impairments was present, irrespective of progress of time. This finding suggests that motor recovery is influenced, in a direct manner, by the recovery of somatosensory impairment.

Meyer et al^50^ studied somatosensory recovery in 32 patients after stroke in the first week and at 26 weeks poststroke. They found only very low correlations between the FM-UE and the subdomains of the EmNSA-UE in the first week poststroke (*r* = −0.03 to −0.14), whereas low to moderate correlations were found at 26 weeks poststroke (*r* = 0.02 to 0.27).^50^ We found, in line with these results, that severe baseline somatosensory impairment does not necessarily prevent spontaneous motor recovery as hypothesized but rather that recovery of somatosensory impairment is required for achieving full motor recovery.

### Differences in Mechanisms, Based on Between- and Within-Subject Effects

The hybrid association model showed that the within-subject effect, which represents the progress of time after stroke and, thus, spontaneous neurobiological recovery, is influenced by factors such as stroke severity. However, we found a clear between-subject effect in the association between motor and somatosensory impairments, which remained significant after correcting for covariates and progress of time. This result reflects the same general concept as was captured in the analyses using motor recovery pattern subgroups based on cutoff grouping variables. Somatosensory impairment affects motor recovery, which supports an underlying mechanism consistent with processes of learning-dependent plasticity.

Although the hybrid model does not give insight into the existence of subgroups of recoverers and non-recoverers, findings do confirm that the association between motor and somatosensory impairments varies between patients with different motor recovery patterns. Note that using a hybrid model may circumvent inherent problems of defining cutoff values in small groups of patients and may, therefore, be recommended as an instrument to separate the within-subject variance from the between-subject variance in explaining neurobiological recovery in repeated measurement designs.^48^

### Limitations

The EmNSA-UE was used to measure somatosensory impairment, as has been recommended, because of its good to excellent reliability.^2,37^ The standardized response mean of the revised NSA has a wide range, from 0.34 to 0.83, depending on the subdomain.^51^ The smallest detectable change or minimal clinically relevant difference of the EmNSA has, however, not been determined. The EmNSA-UE is a broad measure focusing on detection of impairments in the primary somatosensory modalities using a subjective ordinal 3-point scale and does not evaluate somatosensory discrimination, such as tactile 2-point discrimination. It can be hard to obtain an accurate and valid score for patients with cognitive or attention impairments. Hence, we assumed a liberal measurement error of at least 2 points when defining somatosensory impairments.

Although multiple relevant covariates have been taken into account in this study, we could not correct for lesion volume or location in our association model because this information was not available. The results from our study, however, suggest that the association between the recovery of motor and somatosensory impairments is based on more than the close anatomical distance of somato-motor brain areas, the overlap of the lesion in metabolism-dependent systems, and the recovery of penumbral tissue.^52,53^ Type of treatment was also not explicitly accounted for as a potential covariate in the present study, although there is no evidence for a confounding effect.^32^

### Future Directions

Although the underlying neurophysiological mechanisms for motor and somatosensory impairments could not be causally linked in the current study, our results do provide direction for future research. As has been shown in animal models, reduced somatosensory input compromises learning-dependent plasticity.^28-31^ The absence of recovery of somatosensory impairment could potentially compromise learning-dependent plasticity after stroke, resulting in inferior motor recovery. Recovery of sensorimotor impairment after stroke is associated with changes in resting-state functional connectivity in humans and rodents.^54,55^ Hakon et al^56^ recently showed, in an animal model of stroke, that multisensory stimulation through exposure to an enriched environment improves tactile-proprioceptive function and resting-state functional connectivity in mice, as compared with those housed in a standard environment.^56^ Clinically, the present findings suggest that somatosensory retraining could be beneficial, in particular, for those patients who show incomplete motor recovery poststroke, as was suggested in a recent systematic review of Turville et al.^57^ The evidence regarding the effectiveness of available somatosensory retraining programs on sensorimotor function is currently, however, limited.^57-59^ In this vein, it is important to highlight that several items on the FM-UE, like the pincer, spherical grasp, and cylindrical grasp can be considered tasks that depend on sensorimotor function^12^ and that achieving a full FM-UE score will depend on optimal sensorimotor function. Animal models in which the somatosensory and visual impairments are selectively lesioned may give new insights into whether rehabilitation interventions might be able to interact with motor recovery via learning-dependent plasticity.^9,24^

Clinical practice would highly benefit from measures that can more objectively establish somatosensory function. Therefore, one may consider neuroimaging^60^ and specifically diffusion tensor imaging to determine the intactness of structural pathways after stroke.^18^ Neurophysiological techniques that target the intactness of the somatosensory system, such as closed-loop identification techniques by applying position perturbations^61-63^ or somatosensory and median nerve stimulation to the affected arm,^64^ may be more precise ways to test the integrity of somatosensory pathways after stroke than clinical measures.^62-64^ However, the added prognostic value of these noninvasive techniques above clinical testing alone needs further investigation within the first days poststroke.

Imaging parameters of the lesion are needed in future studies to corroborate our current results, which are based on clinical measures of impairment. One of the few imaging studies investigating somatosensory impairment reported a greater lesion load in the corticospinal tracts of patients (n = 32) with impaired ability to perceive a somatosensory stimulus (eg, touch, pressure) at 4 to 7 days poststroke; yet all the patients showed full recovery of this somatosensory modality at 26 weeks poststroke.^18^ However, the authors did not study the relationship with motor recovery. Longitudinal imaging studies relating functional connectivity patterns to motor and somatosensory impairment and recovery are needed to provide more insights into connectional diaschisis and network changes in poststroke recovery.^65^

Recently, Hope et al^66^ highlighted that the 70% proportional recovery rule may be mathematically inflated. The proportional recovery model is also vulnerable for ceiling effects and may, therefore, give a too optimistic impression of the predictability of outcomes.^66^ Prognostic mixture models not suffering from mathematical inflation, may be a next step to improve early individual clinical decision making at stroke units.^67^ Beyond this discussion,^68^ our results indicate that somatosensory recovery is important to explain variability in the percentage of motor recovery, specifically in the subgroup of recoverers. Our results are a first step toward pinpointing factors that may interfere with and/or prevent spontaneous motor recovery in patients early after stroke. Understanding of these factors, such as somatosensory impairment, is needed to develop strategies to optimize quality of movement after stroke.^69^
